# How to Design Integrated Strategies to Improve Healthcare Quality Whilst Containing Healthcare Costs? A Response to the Recent Commentaries

**DOI:** 10.34172/ijhpm.2022.8325

**Published:** 2023-11-29

**Authors:** Erik Wackers, Niek Stadhouders, Anthony Heil, Simone van Dulmen, Patrick Jeurissen

**Affiliations:** ^1^Radboud University Medical Center, Radboud Institute for Health Sciences, IQ healthcare, Nijmegen, The Netherlands; ^2^Ministry of Health, Welfare, and Sport, The Hague, The Netherlands

 We are pleased to read three thoughtful commentaries in response to our scoping review entitled “Hospitals bending the cost curve with increased quality.”^1-4^ We respond to their considerations in this correspondence. Our scoping review collected sparse evidence on integrated hospital strategies to contain costs whilst increasing quality, summarizing main lessons from 19 case studies in scientific and grey literature between 1989 and 2019. We found eleven themes that hospitals should take into account seeking to contain cost and increasing quality of care: strategy, leadership, finances, engagement, projects, culture, support, reorganization, data collection, skill development, and communication. The three commentaries shed light on: (1) why so few case studies were found, (2) what hospitals can learn from these case studies, and (3) what governments can do to support these strategies. In response to these commentaries, we would like to summarize these points and provide our considerations.

## What Is the Best Strategy to Improve Quality and Reduce Costs?

 One of our main findings is that there is no blueprint. This is stipulated by Bal and Wallenburg, who point out that any complex organizational intervention is likely adapted to its local context, and adjusted along its implementation.^1^ Strategic adaptability may be an important precondition for success. However, this does not imply that hospitals cannot learn from others’ experience. It implies that our lessons are general by definition and require further translation to the local context. Bal and Wallenburg also question how our eleven preconditions are related, and whether all are equally important or whether all should be pursued.^1^ They probably are not all equally important and interrelations are not always clear. In line with their view, we would welcome more theoretical work on the system-wide mechanisms between quality improvement and cost containment. Unfortunately, few of the 19 case studies we were able to include provided sufficient detail to study contextual interrelationships. This would advocate work on standardized scientific descriptions of integrated hospital strategies.^1^

## When Is a Strategy Considered Successful?

 Pai points to the fact that relations between quality and costs are spurious and that low cost hospitals may actually also perform less on quality of care. In many cases higher quality needs to be fed with more resources. However, in a substantial number of cases better quality of care does come with lower expenses, for example the reduction of complication through safety procedures in the operation theatre. These are the pathways that our scoping review wanted to get a grip on. Pai also observes the high heterogeneity in quality measures employed in our included case studies.^3^ Healthcare quality is notoriously difficult to measure and to actually demonstrate success is challenging.^5^ Perhaps this is the reason that data monitoring and Information and Communication Technology infrastructure is a predominant factor for success.^3^ The same holds true for costs: it is actually quite challenging to define costs. For example, a strategy to contain total hospital costs by reducing unnecessary treatments may actually increase the cost per patient.^6^ From the hospital perspective, a strategy may be considered successful if costs decline while reimbursements or budgets remain the same, as this would imply additional margins. This may be challenging in itself when the correlation between hospital costs and reimbursements is limited, as is often the case.^7^ But for policy-makers, bending the cost curve implies reductions in hospital budgets/reimbursements. While in theory a shared savings model could pursue both aims, in practice this is challenging — and risky — for hospitals.^6,8^ For example, by demonstrating success in terms of cost containment, a hospital signals a potential for additional cost cutting by payers.

## How to Retain Physician Engagement During Implementation?

 Demonstrating strategy success is also critical to retain employee engagement; strategies require significant efforts of healthcare workers, and it can be rewarding to find that those efforts pay off. However, it is challenging to retain physician support when economic and clinical considerations misalign^9^: cost containment often requires staff reductions, as this is the bulk of hospital spending.^6,10^ Strong leadership is critical to retain employee engagement, especially when workload increases while staff is reduced.^3^

## What Could Policy-Makers Do to Stimulate Bottom-Up Development of Integrated Hospital Strategies?

 While policy-makers cannot enforce upon hospitals to implement a strategy to improve quality while containing costs, governments could improve the health system climate to be more beneficial to the development of these initiatives, as Wuebker points out.^2^ For example, broad outcome-based payment systems may support integrated strategies.^11^ Furthermore, an integrated hospital strategy to improve quality whilst containing costs could be profitable in a competitive environment, when patients can be directed towards the most efficient provider. The apparent paucity of these strategies suggests that competition in the hospital sector is currently not sufficient to reward hospitals for improving quality while retaining costs. Two major preconditions; (1) that patients trust payers to direct them to the most efficient hospital (institutional trust) and; (2) that patients are willing to trade off quality and costs, may not be fulfilled currently.^12^ This suggests that a hospital strategy of high quality and high costs may be easier to accomplish, but also more rewarding for important hospital stakeholders, including its workers. This may partly explain, as Wuebker points out, why private for-profit hospitals and clinics seek for more opportunistic strategies to attract profits.^13,14^

 While we acknowledge that a competitive hospital environment may support increasing quality of care and reducing costs, it may be challenging to set the parameters just right. Besides, hospitals may also strongly respond to nonfinancial competitive incentives. One powerful impulse is the prospect of being a front-runner in healthcare. Receiving praise and positive attention for the hospitals accomplishments can be sufficiently rewarding, and here governments can bring some support to the table. For example, the presentation of a positive case study may provide a competitive benchmark for other hospitals.

## Concluding Remarks

 An integrated hospital strategy to contain costs whilst increasing quality may be uncertain, risky and potentially financially less rewarding than alternative strategies. The bottom-up, context-dependent aspects of the strategy limits broad implementation. However, successful case studies may still inspire and motivate other hospitals to follow suit. Governments could enhance such spillover effects by supporting and showcasing successful hospitals. To fully grasp the complexity of the strategic implementation of containing costs while increasing quality, and shed light on the role of context and interrelatedness of success factors, additional efforts to research case studies in a standardized scientific framework should be promoted.

## Ethical issues

 Not applicable.

## Competing interests

 Authors declare that they have no competing interests.
